# Draft Genome Sequence of *Mycobacterium houstonense* Strain ATCC 49403^T^

**DOI:** 10.1128/genomeA.00443-16

**Published:** 2016-05-26

**Authors:** Anthony Levasseur, Shady Asmar, Catherine Robert, Michel Drancourt

**Affiliations:** Aix-Marseille Université, URMITE, CNRS, UMR 7278, IRD 198, Faculté de Médecine, Marseille, France

## Abstract

*Mycobacterium houstonense* is a nontuberculous species rarely responsible for human infection. The draft genome of *M. houstonense* ATCC 49403^T^ comprises 6,451,020 bp, exhibiting a 66.96% G+C content, 5,881 protein-coding genes, and 65 predicted RNA genes.

## GENOME ANNOUNCEMENT

Refined taxonomic evaluation of the *Mycobacterium fortuitum* third biovariant complex led to the delineation of *Mycobacterium houstonense* as a new species (1). *M. houstonense* is represented by only two isolates from human sources in the United States, including one face wound isolate from Houston, TX, hence the name given to this species (1). Accordingly, the sources of human infection remain unknown, but sequences closely related to those of *M. houstonense* have been detected in consumed fishes (2). Moreover, *M. houstonense* is one of the nontuberculous *Mycobacterium* species containing the *erm* gene, which supports resistance to macrolides (3). It is therefore of medical and general interest to further describe the genome of this species, and we performed whole-genome sequencing of the *M. houstonense* ATCC 49403^T^ strain. Genomic DNA was isolated from *M. houstonense* grown in MGIT Middlebrook liquid culture (Becton, Dickinson, Le Pont-de-Claix, France) at 37°C in a 5% CO_2_ atmosphere. *M. houstonense* genomic DNA was then sequenced in 2 Illumina MiSeq runs (Illumina, Inc., San Diego, CA) using a 6.9-kb mate-paired library. Reads from Illumina were trimmed using Trimmomatic (4) and assembled using Velvet (version 1.2.03) (5). Contigs were combined together by SSPACE version 2 (6), Opera version 2 (7) helped by GapFiller version 1.10 (8), and homemade tools in Python to refine the set. Finally, the draft genome of *M. houstonense* strain ATCC 49403^T^ consists of 27 scaffolds and 197 contigs containing 6,451,020 bp. The G+C content of this genome is 66.96%. Noncoding genes and miscellaneous features were predicted using RNAmmer (9), ARAGORN (10), Rfam (11), PFAM (12), and Infernal (13). Coding DNA sequences (CDSs) were predicted using Prodigal (14), and functional annotation was achieved using BLAST+ (15) and HMMER3 (16) against the UniProtKB database (17). The genome was shown to contain at least 5,946 predicted RNAs, including 6 rRNAs (2 genes are 5S rRNA, 3 genes are 16S rRNA, and 1 gene is 23S rRNA) and 59 tRNAs. A total of 5,881 identified genes yielded a coding capacity of 5,222,064 bp (coding percentage, 80.94%). Among these genes, 4,813 (81.84%) were found to be putative proteins, and 1,068 (18.16%) were assigned as hypothetical proteins. Moreover, 3,338 genes matched a least one sequence in the Clusters of Orthologous Groups database (18, 19) with BLASTP default parameters. *In silico* DNA-DNA hybridization (DDH) (20) was performed with 23 reference genomes selected on the basis of their 16S rRNA gene proximity with *M. houstonense*. The *M. houstonense* genome was locally aligned 2-by-2 using the BLAT algorithm (21, 22) against each one of the 23 selected genomes, and DDH values were estimated from a generalized linear model (23). The DDH (value, ≥25%) was 29.9% (±2.40%) for *Mycobacterium fortuitum* CT6, 29.7% (±2.45%) for *Mycobacterium nonchromogenicum*, and 24.8% (±2.40%) for *Mycobacterium mageritense.*

### Nucleotide sequence accession number.

The *M. houstonense* ATCC 49403^T^ strain genome sequence has been deposited at EMBL under the accession no. FJVO00000000.

## References

[1] Schinsky MF, Morey RE, Steigerwalt AG, Douglas MP, Wilson RW, Floyd MM, Butler WR, Daneshvar MI, Brown-Elliott BA, Wallace RJ Jr, McNeil MM, Brenner DJ, Brown JM 2004 Taxonomic variation in the *Mycobacterium fortuitum* third biovariant complex: description of *Mycobacterium boenickei* sp. nov., *Mycobacterium houstonense* sp. nov., *Mycobacterium neworleansense* sp. nov. and *Mycobacterium brisbanense* sp. nov. and recognition of *Mycobacterium porcinum* from human clinical isolates. Int J Syst Evol Microbiol 54:1653–1667. doi:10.1099/ijs.0.02743-0. 15388725

[2] Lorencova A, Klanicova B, Makovcova J, Slana I, Vojkovska H, Babak V, Pavlik I, Slany M 2013 Nontuberculous mycobacteria in freshwater fish and fish products intended for human consumption. Foodborne Pathog Dis 10:573–576. doi:10.1089/fpd.2012.1419. 23614799

[3] Nash KA, Andini N, Zhang Y, Brown-Elliott BA, Wallace RJ Jr. 2006 Intrinsic macrolide resistance in rapidly growing mycobacteria. Antimicrob Agents Chemother 50:3476–3478. doi:10.1128/AAC.00402-06. 17005837 PMC1610084

[4] Lohse M, Bolger AM, Nagel A, Fernie AR, Lunn JE, Stitt M, Usadel B 2012 RobiNA: a user-friendly, integrated software solution for RNA-Seq-based transcriptomics. Nucleic Acids Res 40:W622–W627. 22684630 10.1093/nar/gks540 PMC3394330

[5] Zerbino DR, Birney E 2008 Velvet: algorithms for *de novo* short read assembly using de Bruijn graphs. Genome Res 18:821–829. 18349386 10.1101/gr.074492.107 PMC2336801

[6] Boetzer M, Henkel CV, Jansen HJ, Butler D, Pirovano W 2011 Scaffolding pre-assembled contigs using SSPACE. Bioinformatics 27:578–579. doi:10.1093/bioinformatics/btq683. 21149342

[7] Gao S, Sung WK, Nagarajan N 2011 Opera: reconstructing optimal genomic scaffolds with high-throughput paired-end sequences. J Comput Biol 18:1681–1691. doi:10.1089/cmb.2011.0170. 21929371 PMC3216105

[8] Boetzer M, Pirovano W 2012 Toward almost closed genomes with GapFiller. Genome Biol 13:R56. doi:10.1186/gb-2012-13-6-r56. 22731987 PMC3446322

[9] Lagesen K, Hallin P, Rødland EA, Staerfeldt HH, Rognes T, Ussery DW 2007 RNAmmer: consistent and rapid annotation of ribosomal RNA genes. Nucleic Acids Res 35:3100–3108. doi:10.1093/nar/gkm160. 17452365 PMC1888812

[10] Laslett D, Canback B 2004 ARAGORN, a program to detect tRNA genes and tmRNA genes in nucleotide sequences. Nucleic Acids Res 32:11–16. doi:10.1093/nar/gkh152. 14704338 PMC373265

[11] Griffiths-Jones S, Bateman A, Marshall M, Khanna A, Eddy SR 2003 Rfam: an RNA family database. Nucleic Acids Res 31:439–441. doi:10.1093/nar/gkg006. 12520045 PMC165453

[12] Punta M, Coggill PC, Eberhardt RY, Mistry J, Tate J, Boursnell C, Pang N, Forslund K, Ceric G, Clements J, Heger A, Holm L, Sonnhammer EL, Eddy SR, Bateman A, Finn RD 2012 The Pfam protein families database. Nucleic Acids Res 40:D290–D301. doi:10.1093/nar/gkr1065. 22127870 PMC3245129

[13] Nawrocki EP, Kolbe DL, Eddy SR 2009 Infernal 1.0: inference of RNA alignments. Bioinformatics 25:1335–1337. doi:10.1093/bioinformatics/btp157. 19307242 PMC2732312

[14] Hyatt D, Chen GL, Locascio PF, Land ML, Larimer FW, Hauser LJ 2010 Prodigal: prokaryotic gene recognition and translation initiation site identification. BMC Bioinformatics 11:119. doi:10.1186/1471-2105-11-119. 20211023 PMC2848648

[15] Camacho C, Coulouris G, Avagyan V, Ma N, Papadopoulos J, Bealer K, Madden TL 2009 BLAST+: architecture and applications. BMC Bioinformatics 10:421. doi:10.1186/1471-2105-10-421. 20003500 PMC2803857

[16] Eddy SR 2011 Accelerated profile HMM searches. PLoS Comput Biol 7:e1002195. doi:10.1371/journal.pcbi.1002195. 22039361 PMC3197634

[17] The UniProt Consortium 2011 Ongoing and future developments at the universal protein resource. Nucleic Acids Res 39:D214–D219. doi:10.1093/nar/gkq1020. 21051339 PMC3013648

[18] Tatusov RL, Galperin MY, Natale DA, Koonin EV 2000 The COG database: a tool for genome-scale analysis of protein functions and evolution. Nucleic Acids Res 28:33–36. doi:10.1093/nar/28.1.33. 10592175 PMC102395

[19] Tatusov RL, Koonin EV, Lipman DJ 1997 A genomic perspective on protein families. Science 278:631–637. doi:10.1126/science.278.5338.631. 9381173

[20] Richter M, Rosselló-Móra R 2009 Shifting the genomic gold standard for the prokaryotic species definition. Proc Natl Acad Sci USA 106:19126–19131. doi:10.1073/pnas.0906412106. 19855009 PMC2776425

[21] Kent WJ 2002 BLAT—the blast-like alignment tool. Genome Res 12:656–664. doi:10.1101/gr.229202. Article published online before March 2002. 11932250 PMC187518

[22] Auch AF, Von Jan M, Klenk HP, Göker M 2010 Digital DNA-DNA hybridization for microbial species delineation by means of genome-to-genome sequence comparison. Stand Genomic Sci 2:117–134. doi:10.4056/sigs.531120. 21304684 PMC3035253

[23] Meier-Kolthoff JP, Auch AF, Klenk HP, Göker M 2013 Genome sequence-based species delimitation with confidence intervals and improved distance functions. BMC Bioinformatics 14:60. doi:10.1186/1471-2105-14-60. 23432962 PMC3665452

